# Beyond Transplantation: Engineering Neural Cell Therapies and Combination Strategies for Spinal Cord Repair

**DOI:** 10.3390/brainsci16010113

**Published:** 2026-01-21

**Authors:** Lyandysha V. Zholudeva, Dennis Bourbeau, Adam Hall, Victoria Spruance, Victor Ogbolu, Liang Qiang, Shelly Sakiyama-Elbert, Michael A. Lane

**Affiliations:** 1 Gladstone Institutes, San Francisco, CA 94158, USA; 2 Department of Physical Medicine and Rehabilitation, MetroHealth Medical Center, Cleveland, OH 44109, USA; 3 Louis Stokes Cleveland Department of Veterans Affairs, Cleveland, OH 44106, USA; 4 Department of Neurobiology and Anatomy, Drexel University, Philadelphia, PA 19129, USA; 5 Spruance and Associates, Jacksonville, FL 32207, USA; 6 Department of Bioengineering, University of Washington, Seattle, WA 98195, USA

**Keywords:** cell transplantation, cell engineering, combination therapy, spinal cord injury

## Abstract

Spinal cord injury (SCI) remains one of the most formidable challenges in regenerative medicine, often resulting in permanent loss of motor, sensory, and autonomic function. Cell-based therapies offer a promising path toward repair by providing donor neurons and glia capable of integrating into host circuits, modulating the injury environment, and restoring function. Early studies employing fetal neural tissue and neural progenitor cells (NPCs) have demonstrated proof-of-principle for survival, differentiation, and synaptic integration. More recently, pluripotent stem cell (PSC)-derived donor populations and engineered constructs have expanded the therapeutic repertoire, enabling precise specification of interneuron subtypes, astrocytes, and oligodendrocytes tailored to the injured spinal cord. Advances in genetic engineering, including CRISPR-based editing, trophic factor overexpression, and immune-evasive modifications, are giving rise to next-generation donor cells with enhanced survival and controllable integration. At the same time, biomaterials, pharmacological agents, activity-based therapies, and neuromodulation strategies are being combined with transplantation to overcome barriers and promote long-term recovery. In this review, we summarize progress in designing and engineering donor cells and tissues for SCI repair, highlight how combination strategies are reshaping the therapeutic landscape, and outline opportunities for next-generation approaches. Together, these advances point toward a future in which tailored, multimodal cell-based therapies achieve consistent and durable restoration of spinal cord function.

## 1. Introduction

Cell transplantation has long been considered a promising approach for repairing the injured or diseased central nervous system, and with advances in stem cell technology it is re-emerging as a leading treatment strategy [1,2,3,4]. In particular, transplantation of neural (neuronal and glial) stem and progenitor cells is proving to be an effective and translationally viable strategy for spinal cord repair. A common goal among all neural transplantation studies is to provide the ‘building blocks’ capable of contributing to repair and facilitating functionally relevant recovery [2]. Over the past four decades, the field of spinal cord repair has evolved from transplanting undifferentiated fetal spinal cord tissue [5,6,7,8,9,10,11,12,13,14,15] to using pluripotent stem cells (PSCs) [16,17,18,19,20,21,22], and now to employing well-defined donor cell populations with specific phenotypes [21,23]. These advances have been enabled by a convergence of developmental biology and cellular and genetic engineering, along with a deeper understanding of circuit-level plasticity in both the intact and injured nervous system.

Most pre-clinical evaluations of neural cell transplantation for spinal cord repair have relied on small animal (mouse or rat) models of cervical or thoracic contusion, compression, transection, or to a lesser extent combinations of these, each of which creates distinct injury environments that influence donor survival, axon extension, and functional recovery. These models differ markedly in inflammation, tissue sparing, and chronic lesion architecture [24,25], which must be considered when comparing between studies, when scaling up for larger animal models, and even more so when considering translation for human SCI. Understanding how injury type and severity, cell dose, site of delivery, treatment timing and outcome measures used to assess recovery differ within each model is essential for closing the translational gap [26].

This review synthesizes current advances in donor cell engineering, transplantation strategies, and integrative combination therapies to outline how emerging approaches are reshaping the future of neural repair. The literature synthesized in this review was identified through broad PubMed and Google Scholar searches encompassing all available publication years, using combinations of terms including: “spinal cord injury”, “cell transplantation”, “genetic engineering”, “cell engineering”, “neuromodulation,”, “neural stimulation” “combination”, “artificial intelligence”, “machine learning and stem cells”, “biomaterials”, “activity-based therapies”, and “pharmacological interventions.” The authors also sourced information from published textbooks on the relevant subject matter (as cited).

### 1.1. Historical Context for Neural Transplantation Approaches

**Figure 1:**
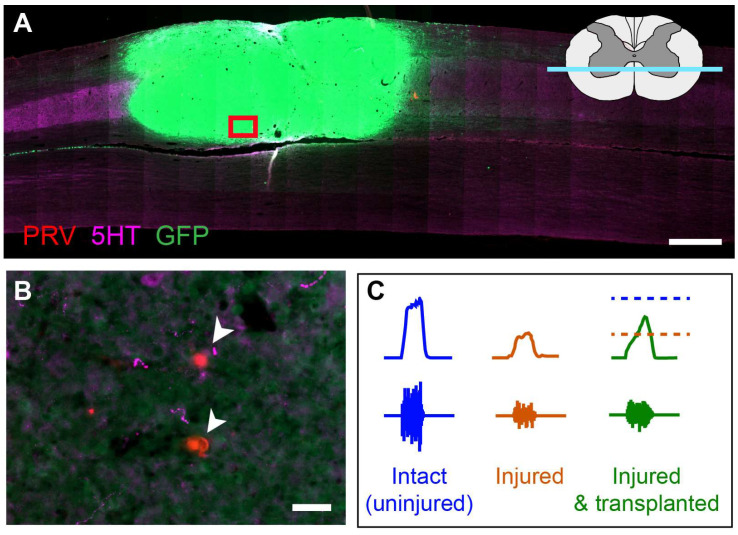
Transplantation of fetal spinal cord tissue into the injured cervical spinal cord of the adult rat. One week following a mid-cervical (C3–C4) contusion injury, when a cystic cavity has begun to form at the injury site, the injured spinal cord was exposed and mechanically dissociated, and green-fluorescent protein (GFP)-tagged, embryonic spinal cord tissue from day 13.5 rat was transplanted directly into the lesion epicenter. One month after transplantation, the phrenic motor network was transneuronally traced from the diaphragm with pseudorabies virus (PRV) and animals underwent terminal diaphragm electromyography to assess phrenic and diaphragm function. EMG recordings were compared to those in uninjured animals (intact spinal cord) and in animals that were injured but not treated. Spinal cord tissues were perfuse-fixed, sectioned horizontally (longitudinal) and processed for immunohistochemistry against PRV, serotonergic axons (5-HT) and GFP. For detailed methods, refer to Spruance et al. [15]. (**A**) Longitudinal section (40 micron, rostro-caudal: left to right) through the cervical spinal cord showing a neural progenitor cell graft (GFP, green) within the lesion cavity. Donor axons extend rostrally and caudally into the host spinal cord. Host serotonergic axons (5-HT, magenta) and pseudorabies virus (PRV, red) labeling identify long-distance supraspinal inputs capable of engaging donor neurons. The inset (red box) marks the region shown at higher magnification in (**B**). (**B**) PRV-positive neuronal somata (arrowheads) within the donor graft, indicating transsynaptic labeling and evidence of host-to-donor circuit connectivity. Labeled serotonergic axons surround these PRV-labeled neurons. (**C**) Representative diaphragm EMG traces showing activity in intact (blue), injured (orange), and injured + transplanted (green) animals. Transplanted animals exhibit partial restoration of phrenic output, demonstrating the functional improvement with transplantation. Scale bars: A = 500 µm, B = 50 µm.

Much of what we have learned about neural transplantation was pioneered by early studies with developing ‘fetal’ spinal cord tissue transplants (Figure 1). Transplanted neural precursor cells obtained from acutely dissected fetal (or more accurately embryonic) spinal cord tissue (FSC) [5,6,7,8,9,10,11,12,13,14], its more selected in vitro expanded counterpart (lineage-restricted neural progenitor cells (NPCs) devoid of extracellular and non-neural components) [27,28,29,30,31,32,33], or even neural stem cells (NSCs) [34,35,36], have been shown to survive, mature, and integrate with the injured adult spinal cord to some extent, often resulting in improved functional outcomes [37,38,39,40]. While FSC or NPC transplants provide evidence of host-donor-host synaptic integration, the degree of integration differs markedly across recipients. Important considerations for donor integration are (1) the time post-SCI that donor cells/tissues are delivered the spinal cord, (2) the site of delivery (lesion epicenter, or rostral or caudal to injury), and (3) the donor cell phenotype(s).

### 1.2. Timing of Treatment

**Figure 2:**
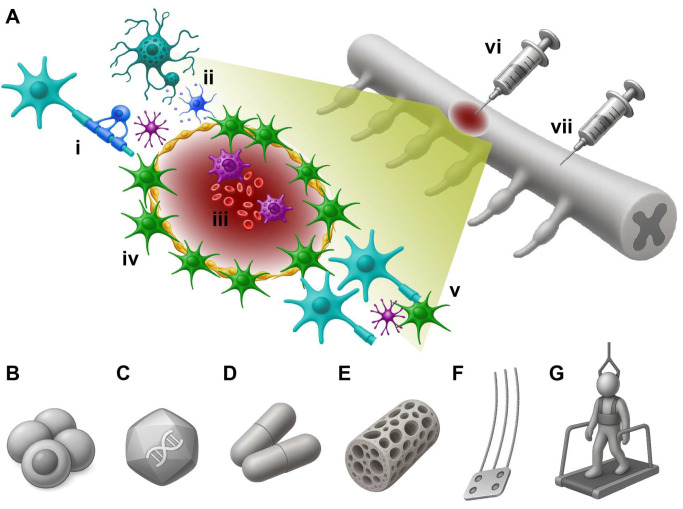
**Cellular and molecular consequences of traumatic spinal cord injury.** (**A**) Schematic representation of the spinal cord after traumatic injury. In most mammalian species, a contusion or compression/crush injury is followed by bleeding into the lesion epicenter and progressive degeneration, resulting in cystic cavitation. (**i**) Neurons (cyan) rostral to injury and their myelinating oligodendrocytes (blue) are not necessarily directly impacted by initial injury. Axons extending into the lesion site and their oligodendrocytes, however, can be compromised (axotomy and primary demyelination). (**ii**) If the axons are compromised close to the cell body, the neuron may undergo degeneration, axons may undergo retraction/degeneration and myelinating oligodendrocytes can be subsequently lost (secondary demyelination). As myelin degrades, associated molecules, such as Nogo-A and myelin-associated glycoproteins, act as inhibitory molecules preventing axonal outgrowth. Reactive microglia (purple) and infiltrating immune cells contribute to early inflammation. (**iii**) Hemorrhage and breakdown of the blood–spinal cord barrier results infiltration of red blood cells (red), plasma, and peripheral immune cells (purple), resulting a cytotoxic, cytokine-rich milieu. (**iv**) Reactive astrocytes (green) and infiltrating fibroblasts (yellow) form a fibroglial border that both protects spared tissue and restricts repair. This glial interface, sometimes referred to as a ‘scar’, also releases chondroitin sulphate proteoglycans (CSPGs) that can act on axonal growth cones to inhibit outgrowth. (**v**) Caudal to injury, glial cells migrate toward areas of Wallerian degeneration and secondary demyelination. Glia accumulate around denervated neurons and increase expression of CSPGs. On the right, the schematic highlights a longitudinal view of the injured spinal cord, illustrating opportunities for (**vi**) pharmacological delivery (e.g., drugs, growth factors, enzymes) and (**vii**) neuromodulatory tools (e.g., epidural stimulation electrodes) to modulate donor and host circuitry. (**B**–**G**) Examples of experimental and emerging therapeutic tools that may be used alone or in combination with neural precursor cell transplantation: (**B**) cell–derived treatments, (**C**) viral vectors for genetic modification or circuit-specific targeting, (**D**) pharmacological agents, (**E**) biomaterial scaffolds for structural and trophic support, (**F**) electrical stimulation interfaces, and (**G**) activity-based rehabilitation paradigms (body-weight support treadmill training depicted here). Together, these illustrate the breadth of strategies designed to improve plasticity, tissue repair and functional recovery after SCI. Illustrations were made with ChatGPT 5.1, Adobe Illustrator 2026 v30.0 and Adobe Photoshop 2025 v26.11.0.

The cellular and molecular consequences of traumatic SCI result in an injury environment that is not amenable to regeneration and repair (Figure 2). The pathophysiological phases of SCI can be broadly defined temporally as (i) acute (0–7 days post-injury), characterized by hemorrhage, excitotoxicity, early immune-cell infiltration, and heightened donor cell vulnerability; (ii) subacute (from approximately 1 week to 1 month post-injury), during which there are ongoing cascades of inflammation, with phagocytosis of cell and tissue debris and progressive fibroglial ‘scarring’ at the lesion site, that stabilize over time; and (iii) sub-chronic (~4–8 weeks) and chronic (>8 weeks post-injury), when a mature fibroglial interface has formed and extensive extracellular matrix deposition has occurred, and circuit reorganization plateaus. Notably, these post-injury timelines are primarily based on small-animal preclinical SCI studies, with reviews showing similar evolving pathology in both preclinical and clinical contexts.

Given this evolving pathology, the time post-SCI at which donor cells or tissues are delivered strongly influences graft survival and integration [41]. Acute transplantation capitalizes on an internal milieu more permissive to integration than seen chronically, characterized by less inhibitory extracellular matrix deposition and active endogenous plasticity, but may also expose donor cells to heightened inflammation and secondary injury cascades [2]. Accordingly, acute transplantation paradigms typically aim to treat this inflammation and promote neuroprotection rather than achieve extensive integration. Subacute delivery may strike a balance between reduced toxicity and preserved plasticity, allowing for tissue repair and integration, but may occur beyond the window for neuroprotection. In contrast, chronic transplantation faces an extensive fibroglial interface or ‘scar’ at the lesion site, potential host axon dieback, inhibitory ECM deposition both at the lesion site and caudally around denervated networks, and circuit reorganization, all of which can limit host or donor neurite extension and synaptic integration. Despite these challenges, multiple preclinical and clinical studies have attempted transplantation of neural tissue at chronic stages following SCI, either alone [7,42,43,44,45,46,47,48,49] or in combination with other treatments [50,51,52,53,54,55]. While donor tissue was reported to survive long-term after chronic transplantation, little is known about the relative extent of integration and potential contribution to anatomical or functional improvement. Temporal differences in the host environment likely contribute to the broad variability reported across studies and emphasize the need for strategies that modulate the host environment to support donor integration across time points.

A related consideration is the extent of injury to be treated with transplantation: while a severe injury will present with the greatest challenges for donor cell survival and integration (greater inflammation, host network compromise and axonal dieback), more modest injury and associated tissue/network sparing will have the greatest potential for intrinsic plasticity and may be a less relevant candidate for invasive cell therapy and repair. With this in mind, one of the gaps between pre-clinical and clinical testing of neural cell therapies is that human injuries are extremely heterogeneous and represent a combination of contusion and compression with potential partial laceration, whereas pre-clinical testing typically models only one aspect of these complex injuries to reduce heterogeneity [56]. Moving forward, machine learning-based analysis of patient data may enable identification of injury types that will best respond to cell-based therapies for people living with SCI.

### 1.3. Location and Integration

The anatomical site of donor cell delivery critically shapes transplant integration and effects (Figure 2) [41]. Transplants placed directly within the lesion epicenter have been shown to fill the cystic cavity (when delivered at appropriate doses) and establish robust donor-host appositions, but they are also subject to the greatest mechanical distortion, inflammation, and limited vascular supply. In contrast, while perhaps more translationally challenging, donor cells placed rostral or caudal to the injury integrate within more preserved tissue, encounter fewer inhibitory signals, and may extend axons across host tissue bridges toward the lesion. The choice of location for transplantation therefore influences whether donor cells primarily replace lost tissue, form relay circuits, or provide trophic support to salvage spared pathways. These distinctions are central to interpreting divergent outcomes across studies.

Another consideration is that the extent of integration that might occur acutely after transplantation could change over time. Indeed, long-term studies have shown that graft survival and connectivity continue to evolve [57]. Comparison of FSC transplants at 1, 3, and 12 months post-transplantation revealed similar transplant survival and tissue confluency between host and donor tissue at each time point, but donor-host connectivity decreased over time [57]. This loss of connectivity over time may be due to pruning of synapses associated with transplant and circuit development. Preventing this loss of connectivity may also represent a novel therapeutic approach to improve neural transplant outcomes.

### 1.4. Identity of the Donor Cells

The donor cell phenotype is also a major determinant of therapeutic role and of both the extent and specificity of integration. FSC grafts contain a heterogeneous mixture of all the building blocks of spinal tissue, including neural (neuronal and glial), microglial and vascular progenitors, which can yield cells that generate broad patterns of synaptic connectivity but may introduce variability between preparations. More lineage-restricted neural stem or progenitor cell preparations can generate refined neuronal subsets and glia, but still contain a mixture of fates influenced by intrinsic patterning and the host environment. Furthermore, such preparations can even limit some efficacious donor populations [23]. Phenotype may also influence graft maturation rate, vulnerability to the inflammatory microenvironment, and long-term stability of connections.

More recently, NPCs derived from human PSCs have also been shown to survive, differentiate, and extend axons following transplantation into injured spinal tissue, promoting tissue repair and improving functional outcomes [14,16,18,20,21,58,59,60]. Promising results using induced PSCs (iPSCs) recently led to the launch of the first-in-human clinical trial for SCI in Japan [61,62]. Despite the tremendous advances made in neural cell therapy, functional outcomes remain inconsistent, highlighting two persistent barriers to effective PSC-based spinal repair:

- The need for well-characterized, pro-reparative donor phenotypes [23,63,64,65];
- The challenge of ensuring robust, consistent, and lasting integration between damaged networks and donor cells.

With these limitations in mind, this review highlights strategies for engineering identified pro-reparative cell types (i.e., cell types found to contribute to measurable and significant anatomical repair) and describes how combining cell transplantation with complementary therapies can enhance donor cell survival, differentiation, maturation, and cellular and synaptic integration.

## 2. Designing and Engineering Donor Cells and Tissues for Spinal Cord Repair

Advances in our understanding of the adult mammalian spinal cord, and the consequences of injury (Figure 2), are informing the design of cell-based repair strategies for SCI. Traumatic spinal cord injury in people is typically a combination of (i) contusion, (ii) compression, with (iii) possible partial laceration (as a result of penetrating foreign objects or dislodged bone fragments) [56] and may involve additional retraction and/or root avulsion. With a few exceptions [66,67,68], preclinical models of SCI usually recapitulate only one or two of these injury types [56,69,70,71,72,73,74]. Key lessons from these studies include why regeneration is limited in adults, in contrast to the developing mammalian [74,75,76] or non-mammalian spinal cord [77,78,79,80,81], and which cellular and molecular mechanisms still enable neuroplasticity [56,82,83,84,85,86,87,88,89]. The primary demyelination observed in some instances following traumatic SCI [90], revealed a therapeutic target for delivery of oligodendrocyte precursor cells [91,92,93,94,95,96], which led to the first-in-human stem cell trial [97,98]. While glial cells contribute to the wound healing response and glial interface that develops at the lesion site [99,100,101,102], the discovery that developing astrocytes can promote repair and support axon growth [103,104] identified immature glial cells as a candidate for transplantation to promote repair [105,106,107,108,109,110]. Similarly, microglia have been shown to be promising candidates for spinal cord transplantation, capable of modulating the host immune response to trauma, with varying reports of improved functional outcome [111,112,113,114,115].

Historically, researchers have explored the transplantation of neural tissues from brain, brainstem, and spinal cord. While there has been some experimental merit to using brain or brainstem cell and tissue sources, given the cellular phenotypes they comprise (e.g., monoaminergic [116,117,118,119,120,121,122,123] or cortical neurons [124,125,126,127]), multiple lines of evidence that indicate that spinal cord cells and tissues are a more appropriate donor type than brain/brainstem sources for repairing the injured spinal cord [59,128].

**Figure 3:**
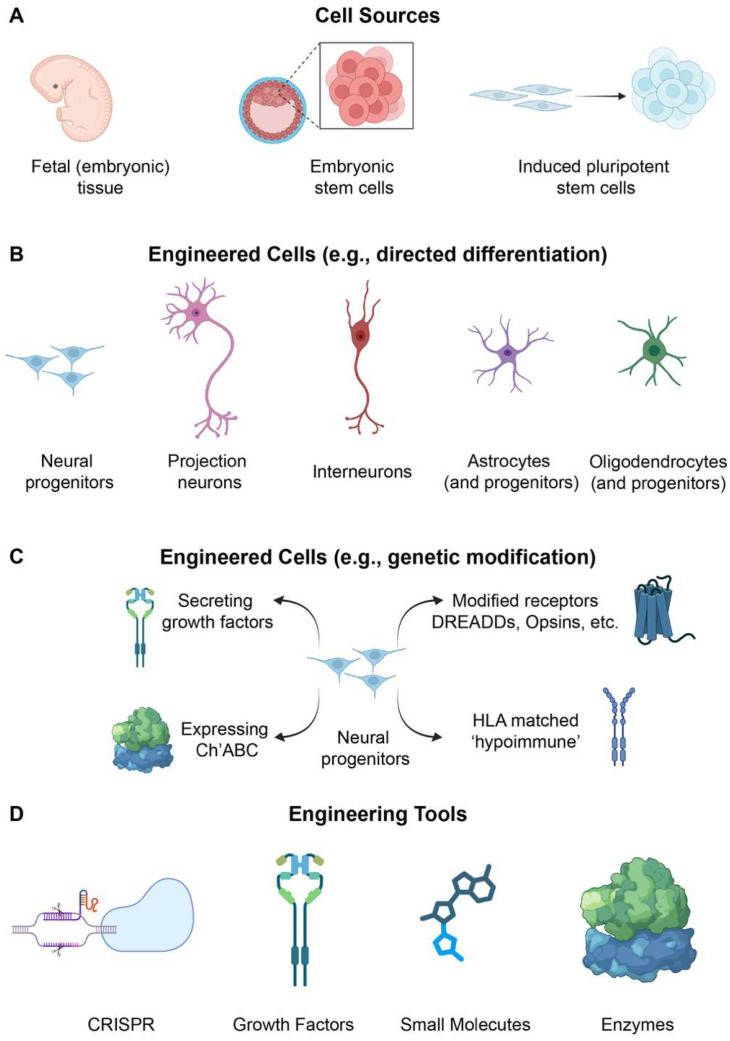
**Stem cell sources, engineered derivatives, and tools for neural repair.** (**A**) Cell sources for transplantation include fetal (embryonic) tissue, embryonic stem cells, and induced pluripotent stem cells, each offering unique advantages and limitations for therapeutic development. (**B**) Through directed differentiation, stem cells can be guided into specific neural lineages, including neural progenitors, projection neurons, interneurons, astrocytes, and oligodendrocytes, thereby enabling tailored approaches for circuit repair and regeneration. (**C**) Engineering strategies further enhance the therapeutic potential of these cells, such as programming secretion of trophic factors, expression of enzymes (e.g., chondroitinase ABC) to remodel the extracellular matrix, introduction of synthetic or modified receptors (e.g., DREADDs, opsins) to enable neuromodulation, or reducing immunogenicity through HLA-matched “hypoimmune” lines. (**D**) A range of engineering tools, including CRISPR-based genome editing, growth factor signaling, small molecules, and enzyme delivery, support the generation of specialized cell products optimized for survival, integration, and function after transplantation. Some components of this figure were created in BioRender (https://BioRender.com/22p1so8, accessed on 24 September 2025).

In some ways, the early pre-clinical efforts transplanting developing/embryonic spinal cord tissues to treat the injured spinal cord remain a ‘gold standard’, as the donor tissues comprise all spinal cellular elements (not just neurons and glia but also vascular endothelial cells and supportive extracellular matrices). Despite some early clinical research using embryonic spinal cord tissue to treat people with SCI [11,12,129,130,131], for ethical and logistical reasons developing embryonic tissues are not a viable ongoing source of donor cells for clinical transplantation. Therefore, carefully controlled, rigorously characterized embryonic stem cell (ESC)- or induced PSC-derived donor cells are far more appropriate as a starting cell source (Figure 3). Human iPSCs can circumvent tissue matching considerations if the cells are derived from the patient’s own cells (e.g., de-differentiated skin fibroblasts). While iPSCs are exempt from many of the ethical concerns associated with using embryonic PSCs, they introduce their own challenges, including genetic stability and reprogramming fidelity. In addition, all stem cell-derived donor cell sources carry tumorigenic risk, and minimizing that risk is an essential step in scaling up donor cell populations for translation. Each of these biosafety considerations underscores the need for rigorous manufacturing, monitoring, and quality control.

As alluded to, the pluripotent starting cell source can be harnessed to engineer virtually any cell type to be used for repair, and historically, spinal cord cells and tissues have been more efficacious for spinal cord repair than brain or brainstem-derived transplants. The question now remains: which cell type to engineer to yield the greatest benefit?

### 2.1. Neurons and Neuronal Progenitors

The spinal cord comprises thousands of neuronal phenotypes that can be broadly divided into projection neurons, spinal interneurons (SpINs), and their progenitors. While the cell bodies of projection neurons are located within the spinal cord, their axons extend outside of the spinal cord. This includes spinal neurons with cell bodies within in the spinal cord with ascending projections to innervate the brain and brainstem, and spinal motor neurons with cell bodies within the cord but extending axons out into the periphery. In contrast, the cell bodies of SpINs reside in the spinal cord and they innervate other neurons in the spinal cord (projection neurons or other SpINs).

The newly appreciated heterogeneity between cervical, thoracic and lumbar spinal cord [132], highlights the need to consider distinct classes of SpINs. Long ascending and descending SpINs that connect distant regions likely exhibit genetic, morphological, electrophysiological, neurochemical and network characteristics that differ from those of short-projecting SpINs restricted to a single spinal region or segment. Regardless, all SpIN populations and projection neurons can be lost after traumatic SCI [133,134,135], and both represent therapeutic targets for transplantation [136,137].

Early attempts at transplanting a particular type of projection neuron—the motor neuron—highlighted two major challenges in this approach: (i) reliable isolation or derivation of bona fide spinal motor neurons from PSCs, and (ii) the difficulty of getting transplanted motor neurons to extend axons long distances to innervate peripheral organs. Although some studies transplanted motor neurons derived from embryonic tissues, or PSC-derived motor-neuron-like cells, directly into the injured spinal cord, these donor cells mainly appeared to provide trophic support or relay functions rather than true re-innervation of muscle [138].

More recently, attention has shifted toward SpINs, the excitatory, inhibitory and modulatory neurons that form intrinsic spinal circuits [41,63,64,65]. SpINs are central to spontaneous and therapeutically mediated functional recovery, and can serve as relay neurons to re-establish new neuronal networks that bypass the injury site [83]. Pre-clinical studies show that neural stem/progenitor cell transplants enriched for defined SpIN subtypes yield superior outcomes [15,21,23,139]. For example, in a rat model of cervical SCI, transplanted NPCs enriched with PSC-derived V2a interneurons (excitatory interneurons) survived, differentiated, and integrated with the injured spinal cord, leading to significantly improved motor function [23]. These findings highlight that targeting specific cardinal SpIN classes—such as excitatory V2a or modulatory V0c SpINs [21,64,140]—can promote effective repair of damaged neural pathways.

Parallel approaches are transplanting primary or PSC-derived brain GABAergic interneuron precursors to rebalance hyperactive autonomic and sensory spinal circuits. These precursors, originally developed for transplantation into epilepsy and pain models, can survive, integrate, and release inhibitory neurotransmitters when transplanted into a mouse model of SCI. Mice receiving these transplants exhibited significantly improved bladder function and a reduction in pain hypersensitivity [141,142,143,144,145]. Collectively, these results highlight SpIN donor cells as an effective cell type for spinal cord repair and restoration of function after SCI, placing them at the forefront of reparative strategies. With ongoing efforts to better understand the contribution of SpIN subtypes to plasticity and recovery, future work will be able to generate regionally matched SpIN subtypes to repair the injured spinal cord and restore diverse spinal functions.

### 2.2. Glia and Glial Progenitors

While a focus on neuronal donor cells remains a central theme in neural transplantation, glial cells have also been long recognized for their ability to modulate the injury environment, provide metabolic support and facilitate neural repair. Glial-restricted progenitors (GRPs) isolated from the embryonic spinal cord can survive after transplantation in the injured spinal cord for weeks, differentiate into oligodendrocytes and astrocytes, and migrate rostrally and caudally. Donor glial progenitors attenuate glial scarring, reduce inflammatory infiltration, enhance angiogenesis and stimulate/support host axon outgrowth [2,146,147,148]. Importantly, the stage of glial development and potential astrocytic phenotype may influence their reparative potential: GRPs differentiated into bone morphogenetic protein-induced astrocytes secrete fewer chondroitin sulphate proteoglycans (CSPGs), promote dorsal column and rubrospinal tract regeneration, and improve motor control, whereas ciliary neurotrophic factor-induced astrocytes deposit more inhibitory matrix and can worsen fine motor recovery [149]. Attention should therefore be paid to the intended purpose of transplanted glial cells and their phenotypic specification, as these factors will determine whether transplants primarily support neuroprotection, immune modulation, or axonal regeneration.

Beyond astrocytes, oligodendrocyte precursor cells (OPCs) have been explored to remyelinate demyelinated axons and restore conduction, with pre-clinical and early-phase clinical studies suggesting these cells can enhance functional recovery [92,93,94,96,150]. For example, human ESC (hESC)-derived OPCs transplanted into adult rats one-week post-SCI survived, differentiated into oligodendrocytes, enhanced myelination, and led to substantial locomotor improvement. In contrast, delayed transplantation of these cells at 10 months post-injury failed to produce benefits [94]. These findings were further explored in a cervical SCI model, where hESC-derived OPCs attenuated lesion pathology, preserved white and gray matter (including motor neurons), improved forelimb function [92] and were confirmed to be safe [96]. These preclinical successes catalyzed the first FDA-approved Phase I clinical trial (GRNOPC1) using OPCs for remyelination in SCI patients. While GRNOPC1 represented a landmark step toward clinical translation, a detailed examination of the trial’s subsequent progression, the economic and regulatory considerations, and the study methods that shaped its trajectory, is beyond the scope of this review. Readers seeking a comprehensive analysis of clinical trial successes and limitations—including why many promising strategies have not advanced despite strong pre-clinical rationale—are referred to Scheuren & Kramer (2024) [151] and Jones et al. (2025) [26]. Previous reviews provide a comprehensive list of clinical trials in spinal cord injury [2] or even more specifically, combinatorial strategies [152].

With advances in directed differentiation from PSCs, the generation of defined oligodendrocyte or astrocyte subtypes provides new opportunities to harness glial biology in combination with neuronal donor cells to promote durable spinal cord repair. As described in the next section, progress in cellular engineering now enables genetic modulation to further boost cells’ reparative potential (e.g., overexpressing trophic factors or suppressing scarring signals). The challenge ahead is to generate glial cells with pro-reparative and regionally specific phenotypes, to optimize their therapeutic potential.

### 2.3. Next-Generation Donor Cells: Genetic Engineering

As the field of cell-based therapies for spinal cord repair matures, there is growing interest in moving beyond unmodified donor cells toward next-generation, genetically engineered cell products. Recent advances in cellular engineering, genome editing (e.g., CRISPR-Cas9), and synthetic biology now allow donor cells to be tailored for enhanced survival, integration, activity modulation, and even immunological compatibility. These enhancements offer new opportunities to overcome biological barriers in the injured spinal cord and extend the therapeutic potential of transplanted cells.

One of the most widely explored genetic modifications to donor cells is the enhanced production of trophic factors or synaptic organizers to promote neuronal survival, axonal growth, and synaptic plasticity. For instance, NPCs engineered to overexpress BDNF, NT-3 [153], GDNF [154,155,156,157], or CPTX [158] have been shown to exhibit improved donor cell survival, axon growth, and synaptic formation, and enhanced integration. A recent notable example includes the transplantation of GDNF-expressing human iPSC-derived NPCs, which showed enhanced axonal growth and functional recovery in a rodent model of SCI [157]. These engineered NPCs illustrate how targeted expression of neurotrophic molecules can significantly boost the therapeutic value of transplanted cells.

Immune rejection remains a major hurdle in allogeneic transplantation, often necessitating long-term immunosuppression to ensure survival, which comes with numerous side-effects that can be detrimental to host health. A potential solution to this problem is transplantation of iPSC-derived cells from a patient’s own tissue sample. While clinical trials are now underway using autologous iPSC donor cells to treat people with Parkinson’s disease [159], there are challenges to rapidly, reliably and consistently obtaining and engineering somatic cells into iPSCs from patients. Instead, most studies transplanting iPSC-derived cells are still using allogeneic cells, including the first-in-human iPSC clinical trial for people with SCI [61,62]. Donor cells can now be engineered to evade immune detection [160]. Strategies include CRISPR-mediated deletion of MHC class I and II genes, or co-expression of immunomodulatory molecules such as PD-L1 or CD47, to generate hypoimmunogenic donor lines. These more universally compatible cell lines offer the possibility of off-the-shelf therapeutic products that minimize the need for immunosuppressive regimens and reduce the risk of donor cell rejection or inflammatory complications. This is a rapidly evolving area of research, and growth is expected in both pre-clinical studies and clinical trials using either autologous iPSCs or hypoimmune allogeneic PSCs in the coming years.

While donor cells are known to migrate and extend neurites into host tissues, they are still confronted with the inhibitory environment of the injured spinal cord (Figure 2), and integration is inconsistent between recipients. While some strategies can target the host to increase trophic factor expression (e.g., activity-based therapies, neural stimulation or gene therapy), donor cells can also be genetically modified to promote greater integration. A number of donor cell types have been modified to target the growth inhibitory cues found within injured tissue. For instance, lineage-restricted precursor cells have been engineered to express chondroitinase (to enzymatically degrade CSPGs), and shown to extend neurites through inhibitory environments in vitro [161]. There have also been efforts to target the inhibitory effects of NogoA by expression of the Nogo-66 receptor antagonist; NEP1-40. While efforts to test this have been limited, evidence suggests that NEP1-40 expression in donor cells supports both neuronal differentiation and neurite extension [162,163].

Beyond improving survival and integration, genetic engineering also enables precise control over the activity of transplanted neurons. Tools such as chemogenetics (e.g., Designer Receptors Exclusively Activatable by Designer Drugs or DREADDs), optogenetics, and sonogenetics allow researchers to activate or inhibit donor neurons in a temporally and spatially precise manner using designer drugs, light, or ultrasound, respectively. For instance, DREADD-expressing human neural stem cells have been transplanted into injured spinal cords in rodent models to evaluate how modulating donor neuron activity influences circuit formation and recovery [164,165]. Similarly, optogenetic transplants allow for the real-time testing of donor cell contributions to behavior or function and provide a research platform for mapping synaptic integration [21,166,167].

Despite their therapeutic promise, each of these engineering strategies presents important translational challenges. CRISPR-based genome editing, although precise, carries risks of off-target modifications, genomic instability, and unintended alterations in transcriptional networks that may impact donor cell safety. Similarly, sustained overexpression of trophic factors or extracellular matrix-modifying enzymes raises concerns regarding long-term stability, regulation, and potential adverse effects such as maladaptive sprouting or disproportionate growth support. Chemogenetic and optogenetic systems introduce additional hurdles, including dependence on exogenous ligands or light-delivery hardware, compatibility with implantable devices, durability of gene expression, and the difficulty of maintaining reliable, spatially restricted control of neuronal activity in a clinical environment. Collectively, these considerations underscore that while engineering donor cells offers opportunities to enhance survival, integration, and circuit remodeling, careful risk assessment and improved regulatory frameworks will be essential for safely advancing these tools towards translation.

## 3. Combination Therapies

Despite the significant promise of cell-based interventions for SCI, it is widely accepted that no single treatment is likely to achieve complete functional restoration. Some cell therapy approaches could be considered combination treatments as they use a cocktail of cell types each with unique therapeutic functions (e.g., neural stem/progenitor cells comprise mixed populations of neurons and glia [2,168], Schwann cells and olfactory ensheathing glia [169,170]). However, mounting evidence suggests that combining cell transplantation with non-cellular interventions (e.g., drugs, biomaterials, neural stimulation or activity-based therapies) offers benefits beyond what cells alone can achieve [52,152,171,172,173].

Combination or combinatorial approaches that pair cell transplantation with other cellular or non-cellular therapies are gaining traction as a means to synergistically enhance outcomes. These adjunctive treatments can (i) support transplanted cells by promoting survival, migration, and integration, thereby making the cell therapy more effective, and/or (ii) offer independent effects (e.g., host tissue neuroprotection, enhance plasticity and host circuit reorganization) to synergistically improve repair and recovery. With a focus on NPC transplantation for spinal cord repair, we now highlight some of the strategies that have been used to improve functional outcomes.

### 3.1. Combining NPCs with Other Donor Cells

**Figure 4:**
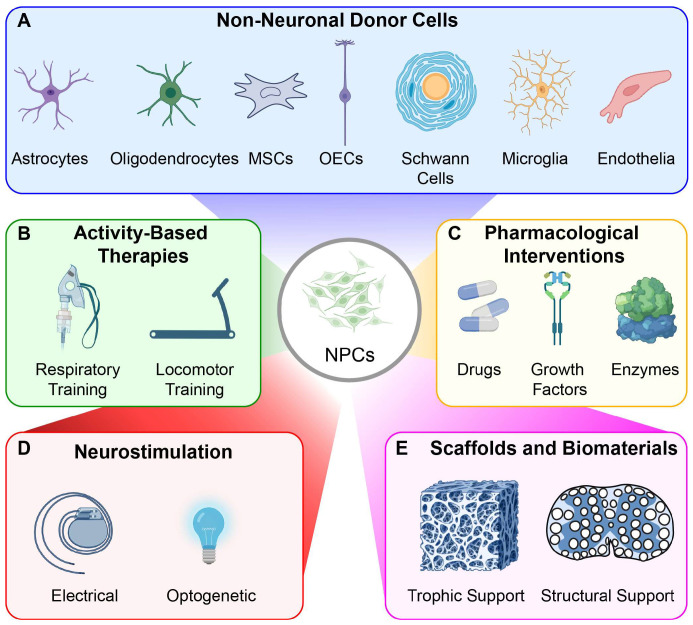
**Complementary strategies to enhance neural progenitor cell (NPC) therapies.** (**A**) Non-neuronal donor cells such as astrocytes, oligodendrocytes, mesenchymal stem cells (MSCs), olfactory ensheathing cells (OECs), Schwann cells, microglia, and endothelial cells provide trophic, structural, or modulatory support and can be combined with NPCs to improve outcomes. (**B**) Activity-based therapies including respiratory and locomotor training promote circuit plasticity and functional recovery, creating an environment conducive to transplant integration. (**C**) Pharmacological interventions such as drugs, growth factors, and enzymes can enhance survival, differentiation, and connectivity of transplanted cells. (**D**) Neurostimulation approaches such as electrical and optogenetic stimulation modulate activity-dependent plasticity and improve transplant-host communication. (**E**) Scaffolds and biomaterials provide both trophic and structural support, improving donor cell survival, guiding axonal growth, and stabilizing transplanted tissue. Some aspects of this figure were created in BioRender (https://BioRender.com/3urywx2, accessed on 24 September 2025).

Combined cell therapy offers a sophisticated strategy to address the complex challenges of SCI. While transplanted NPCs provide both structural and functional benefits in animal models of SCI [2,23,36], their therapeutic potential may be partially constrained by the internal milieu of the injured tissue. This includes dominant glial differentiation, debris that impedes synaptic connectivity, and vascular loss that causes local ischemia and deprives the transplant of metabolic support [174]. To address these barriers, the addition of other cell types has been investigated, including olfactory ensheathing cells (OECs) or Schwann cells to guide axonal growth, mesenchymal stem cells to secrete neuroprotective and trophic factors, and genetically modified M2-like macrophages to clear debris, secrete wound-healing factors, and modulate inflammation [175,176,177,178,179]. Co-transplanting NPCs with multiple cell types may create a synergistic, pro-reparative environment that supports the survival, integration, and functional contribution of transplanted cells, ultimately improving outcomes after SCI (Figure 4).

A vast range of cells can support spinal cord tissue repair and axonal regeneration, such as the ependymal cells derived from the spinal cord central canal [75,180,181,182,183,184]. Studies have shown that co-transplanting NPCs with other cell types, such as mesenchymal stem cells (MSCs), or OECs, can significantly enhance therapeutic outcomes in SCI compared to NPCs alone, underscoring the value of combination strategies [185,186]. The challenge lies in determining the most effective way to combine these cells (e.g., anatomically, temporally). One approach is simultaneous transplantation, delivering NPCs with supporting cells at the same time in the same location [187]. Alternatively, a staged approach can be used, where supporting cells are transplanted first to modulate the injury environment, followed by NPC delivery [185]. Another method involves co-culturing supporting cells with NPCs in vitro, enabling trophic factor exchange and promoting neuronal differentiation and maturation prior to transplantation [188]. However, conventional 2D culture limits cell–cell interactions and functional maturation, as NPCs grown in 2D differ transcriptionally from those in 3D [23]. Using 3D systems such as spheroids, organoids and microtissues allows pre-formation of neuron–glia interactions along with other supporting cells, potentially improving graft integration and long-term functional recovery after transplantation.

Recent advances in stem cell biology have enabled the generation of organoids, self-organizing 3D structures that model specific CNS regions [189]. While cortical organoids are well established, protocols for spinal cord microtissues and organoids are only recently emerging [190,191,192]. These systems incorporate a host of diverse SpINs, motor neuron progenitors and glial progenitors, providing the building blocks for the injured spinal cord to make new circuits as well as support myelination and donor tissue integration.

A major hurdle for spinal cord organoids is vascularization. Recent work has demonstrated that incorporating vasculature into neural organoids improves survival, maturation, and tissue complexity, resulting in enhanced progenitor proliferation, reduced hypoxia, and development of intact neurovascular units with barrier-like features [193,194,195,196]. In addition to providing the blood supply these tissues need, the vascular network can support axonal growth and may be an important component of integration between host and donor neurons. These studies highlight that vascularization is not merely supportive but actively shapes neural differentiation and circuit formation, underscoring the need for engineered neurovascular units in next-generation spinal cord organoids intended for transplantation.

Given the role of microglia in synaptogenesis, they may facilitate NPC host-donor integration. Accordingly, efforts are being made to incorporate microglia into transplantation protocols to better recapitulate the complex cellular environment of the human nervous system [193,197]. Donor tissue microglia may also exert anti-inflammatory and pro-reparative effects, further enhancing tissue repair and functional recovery after SCI. However, donor microglia may adopt a pro-inflammatory, M1-like phenotype in the injury environment. One possible solution is to genetically program microglia toward a stable M2-like, pro-reparative state prior to transplantation [179]. By integrating NPCs with carefully selected supporting cells and leveraging advanced bioengineered constructs such as vascularized, immune-competent spinal cord organoids, combined cell therapy holds the potential to recreate a functional, pro-reparative microenvironment that overcomes the multifaceted barriers of SCI and drives meaningful, long-lasting neurological recovery.

### 3.2. Combining Non-Cellular Interventions with Neural Cell Transplantation

Much like cell–cell combination therapies, the approach of combining neural cell therapy with non-cellular therapies is grounded in the need for NPCs to have a supportive environment to survive, integrate, and mature into functional circuits [2,41]. For example, the use of neuroprotective drugs may improve the internal milieu of the injured spinal cord to make it more amenable to repair, improving the success of transplanted cells. Delivery of trophic factors (BDNF, NT3) can promote donor cell survival, support migration of donor glia, and encourage outgrowth of donor neurites, depending on when and where they are delivered. Gene therapy has also been used to target the host tissue, as a means to deliver these pharmacological agents, as have biomaterials, with the latter often bringing additional benefits to both the host and donor cells. Activity-based rehabilitation promotes host plasticity and circuit reorganization, which can complement and accelerate the integration of donor neurons [52]. Similarly, neural stimulation (e.g., epidural) can modulate host spinal network excitability and facilitate more focused activity-dependent plasticity, potentially guiding or amplifying the activity and output of transplanted cells. While tests of such combinations are still in their infancy in preclinical models, evidence suggests that these strategies can improve functional outcomes beyond what is typically observed with either intervention alone.

### 3.3. Pharmacological Interventions

Perhaps one of the most widely tested strategies has been the combination of cell transplantation with cytokine delivery for neuroprotection (e.g., trophic factors), to limit host tissue damage and/or promote donor cell survival. Early studies by Bregman et al. found that combining FSC tissue transplants with BDNF and/or NT3 enhanced donor and host cell survival, and promoted neurite growth to and from the transplant, under a variety of injury conditions [198,199,200,201]. Recognizing the neuroprotective and cell guidance properties of these growth factors, researchers started using lentiviral vectors to generate attractive gradients beyond the transplant to coax donor cell outgrowth to host networks [202,203,204,205,206,207,208]. Other studies have attempted to address the concerns surrounding transplant survival and lack of neuronal differentiation by delivering growth factor cocktails with the transplant at the time of injection. These growth factors help enhance survival in severe hemi- or transection injuries, across acute, subacute, and chronic timeframes [14,16,18,58,123,128,166]. Recent iterations of this growth factor cocktail, shown to have comparable efficacy, have been reduced to BDNF, FGF2, VEGF, and a calpain inhibitor to limit apoptosis [52,209,210]. This strategy promotes substantial growth to and from the transplant. Apart from delivering the growth factors at the time of the transplant surgery, groups have also administered growth factors through osmotic pumps that control the degree and timing of growth factor introduction into the system [53,211]. Growth factors are likely to continue to be a critical aspect of many combination therapies going forward, as they can be matched to the needs of the injury and timing of the transplant.

Transplanted cells themselves play an important role in providing factors that mitigate ongoing inflammatory cascades and secondary tissue damage, but they face tremendous rates of cell death acutely post-transplantation [124]. Researchers have directly targeted pro-inflammatory cytokines such as TNF-α [212], and IL-6 [213] to enhance donor survival, differentiation, and motor recovery through enhanced donor-host integration. A more common anti-inflammatory is the tetracycline antibiotic minocycline, which weakens inflammation, dampening the astrofibroglial response to injury and tissue cavitation while improving behavioral recovery alongside NSCs [53,214]. Other recent studies have incorporated anti-inflammatory treatments including curcumin [215,216,217] and erythropoietin [218]. These treatments limit inflammation via widespread antioxidant effects, while erythropoietin also exerts angiogenic activity. Though each of these combination therapies show some enhanced efficacy compared to cell transplant alone, including tissue sparing and improved donor survival, the recovery seen was inconsistent [215,218].

Cyclic AMP (cAMP) and rolipram have been appealing options to pair with cell therapies as they promote integration with host systems through enhancing donor cell activity [219], while simultaneously attenuating inflammation [220]. cAMP’s excitatory properties were initially found to enhance recovery and axon sparing of transplanted NSCs [221]. Rolipram’s ability to inhibit the hydrolysis of cAMP, enabling consistent CNS excitation, has made it a preferred alternative [222]. Rolipram enhances the therapeutic efficacy of numerous transplanted cell types including Schwann cells [223], and OECs [224], but decreased transplant size and serotonergic axon density when used in concert with GRPs [225]. This suggests that activity enhancing drugs should be scrutinized for the proper cellular partner and transplant conditions to ensure robust cell survival and integration.

In conditions where cell survival is preserved and inflammation is manageable, there are still a variety of inhibitory factors in the internal milieu of the injured spinal cord that can limit the integration between donor and host networks. Even though transplanted NPCs have been shown to attenuate CSPGs [226], their expression by both the fibroglial interface at the lesion site and the perineuronal net around some host neurons may still limit the capacity for transplanted neurons to form functional synapses with host networks. However, chondroitinase can degrade CSPGs both at the lesion as well as the perineuronal net surrounding the target host neuron targets, enabling axons to grow through these sites and form synapses [227], making it one of the most widely used pharmacological agents to combine with cells [228,229,230,231,232,233,234,235]. Other drugs promoting neurite outgrowth (or attenuating its inhibition) that have been found to enhance efficacy have included those targeting the Nogo receptor [236], semaphorin 3A inhibition [50], and Rho/ROCK inhibition [237].

Importantly, the method (location and timing) of drug delivery is a major determinant of efficacy. While systemic administration is least invasive and easiest, it risks failing to achieve therapeutic concentrations at the target site (e.g., lesion site, surrounding tissues or transplant) and exposes donor cells to fluctuating levels of inflammatory and trophic signals, contributing to inconsistencies across studies. More targeted approaches, such as intrathecal or intraparenchymal delivery, can increase drug exposure at the target site, but these methods still face challenges such as clearance and/or difficulty maintaining stable local concentrations. These methods also allow for sustained delivery of the drugs over time (e.g., via infusion pumps), but there is a risk of the route of delivery being inadvertently compromised during the time-course of delivery (e.g., the foreign object implanted for delivery can be blocked by cellular debris or scar tissue). Although usually more invasive, biomaterial-based delivery platforms highlighted below offer a promising alternative, allowing for sustained delivery at the target site. Likewise, viral vector systems enable cell-specific or lesion-targeted modulation of the host milieu, and engineered donor cells may eventually provide autocrine and paracrine support without external dosing. Together, these strategies highlight that effective integration of pharmacological agents with neural transplants may rely as much on how drugs are delivered as on which drugs are chosen.

### 3.4. Biomaterials

Biomaterials have emerged as powerful tools in regenerative strategies for SCI [238,239,240,241,242], especially when used in combination with cell transplantation [4]. Being far more than passive carriers, biomaterials can actively influence the fate and function of transplanted cells by mimicking aspects of the native extracellular matrix (ECM), delivering biochemical signals, and even modulating the immune response. They can provide mechanical structure and bioactive cues to donor cells, and spatiotemporal control over the injury environment, both of which are critical for enhancing transplant survival, integration, and reparative efficacy.

The choice of biomaterial composition and structure is central to its ability to support neural repair. Biomaterials designed for drug delivery need to take into consideration the temporal release profile and biocompatibility with spinal tissues. Biomaterials can provide adhesion sites for donor cells to allow integration with host tissues, and need to avoid disrupting host tissue structure through mechanical mismatch. Biomaterial scaffolds must strike a balance between providing mechanical support and accommodating host tissue dynamics in very soft neural tissues. Key design parameters include stiffness, porosity, degradation rate, and topographical features, each of which can influence drug delivery and donor cell adhesion, migration, differentiation, and axonal outgrowth. While natural materials (e.g., collagen, fibrin, hyaluronic acid, chitosan, alginate or combinations [242]) offer inherent biological compatibility and cell adhesion motifs, they may lack tunability in mechanical properties, adhesion sites or degradation time. In contrast, synthetic materials (e.g., PLGA, PEG, self-assembling peptides [242]), which can be tailored to the host environment [243], allow for control over degradation kinetics and mechanical properties but often require ‘biofunctionalization’ to promote donor cell adhesion and survival. Biofunctionalization is the modification of synthetic materials to give them biological properties. For example, while PEG provides excellent mechanical support and can be chemically functionalized to tune material degradation properties, it lacks the binding sites for donor cells. To address this, researchers can incorporate extracellular matrix proteins (or peptides from these proteins) and growth factors [244]. Hybrid materials combine the strengths of natural and synthetic materials to achieve additional therapeutic properties. For instance, while fibrin serves as a useful scaffold material, combining it with extracellular matrix-derived peptides enhances its capacity to support neurite growth [245].

Biomaterials serving as vehicles for the localized, controlled release of drugs that modify the host environment do not necessarily need to provide structural support. This often means that their delivery can be minimally invasive compared to more rigid scaffolds that require invasive surgery (e.g., myelotomy) for implantation. For instance, injectable hydrogels engineered to release trophic factors (e.g., BDNF, NT-3) [246,247], adhesion molecules [248,249,250], or anti-apoptotic agents to enhance cell survival and integration [251], can be readily delivered with cells into the lesion site. These hydrogels often undergo in situ gelation or shear thinning/thickening, conforming to the geometry of the lesion environment and sealing in transplanted cells. Microparticles or nanoparticles can also be co-injected with donor cells, and offer additional control over the release kinetics of therapeutic agents, with a secondary material in the particles regulating release. Such controlled delivery from these materials enables precise temporal modulation of the injury environment, which is especially important in the subacute to chronic phases of SCI when inflammation and secondary degeneration are ongoing. These biomaterials can be engineered to deliver a vast range of drugs that activate specific signaling pathways to (i) guide neuronal differentiation [252,253], or (ii) attenuate inhibitory molecules and create a more permissive host microenvironment for neurite extension and synapse formation [233,234,254]. Some biomaterials have also been engineered to modulate inflammation and prevent immune rejection [255], offering an alternative to immunosuppression.

Biomaterials can also serve as a conduit to neuromodulation [256]. Recent advances in material science have enabled the development of electrically conductive scaffolds, incorporating materials such as graphene, carbon nanotubes, or conductive polymers (e.g., PEDOT:PSS). These scaffolds are designed to support or enhance electrophysiological signaling within the spinal cord, either endogenously or when paired with external neuromodulation techniques (e.g., electrical stimulation). Early studies have demonstrated that these conductive matrices can promote neurite outgrowth, synaptic activity, and regeneration when appropriately stimulated, suggesting a promising avenue for coupling materials with bioelectronic interfaces. Moreover, conductive scaffolds may be particularly effective when combined with optogenetic or chemogenetic donor cells, offering opportunities for closed-loop systems that integrate electrical feedback with engineered cellular responses.

Over the past few decades, an array of pre-clinical studies have combined biomaterials with cell transplantation to treat SCI (reviewed in [173,257]). Injectable hydrogels have been used to deliver human neural stem cells into contused rat spinal cords, improving donor cell survival and differentiation, and motor recovery compared to cell injection alone [257,258,259,260,261,262]. Fibrin-based scaffolds have been used to guide host axon extension into and through donor cell transplants to enhance integration and functional outcomes [14,263,264]. 3D-printed scaffolds, customized to lesion size and shape, have been seeded with human iPSC-derived neural progenitors and neurotrophic molecules to promote long-distance axonal growth and tissue bridging [243]. Vascularized matrices, incorporating endothelial cells and ECM proteins, designed to improve implant perfusion, have been shown to improve spinal cord repair [265]. These examples underscore the synergistic potential of combining biomaterials with cell transplantation, not only to deliver and support donor cells more effectively, but to actively shape the environment in which repair must occur.

While biomaterials provide essential structural and biochemical support to enhance donor cell survival and integration, they remain only one part of the combinatorial toolkit needed to restore function after SCI. Even well-integrated cells must be functionally incorporated into host circuits to contribute meaningfully to recovery. This requires engagement of activity-dependent mechanisms that promote synaptic strengthening, circuit reorganization, and long-term plasticity. Neuromodulation via activity-based therapies (ABTs) or direct stimulation can leverage these principles and have shown promise in guiding the functional integration of transplanted cells. As such, the next generation of combined strategies is increasingly focused on pairing cell transplantation with interventions that harness neuronal activity to drive connectivity and recovery.

### 3.5. Activity-Based Therapies

Neuronal activity is a fundamental driver of synaptic connectivity and circuit refinement. As stated by the well-known Hebbian theory, “cells that fire together, wire together”, repeated and persistent co-activation of neurons leads to the strengthening of their synaptic connections [266]. This activity-dependent synaptic plasticity has been extensively documented in various neural systems, including motor circuits, where it facilitates the reorganization and strengthening of synaptic connections following injury. In the context of SCI, leveraging activity-dependent mechanisms through targeted therapies holds promise for enhancing the functional connectivity in spared pathways, or in the present context, of transplanted neurons with the injured spinal cord. Some studies suggest that combining cell transplantation with ABTs, such as respiratory and locomotor training (e.g., intermittent hypoxia and treadmill training, respectively), may promote the synaptic incorporation of donor neurons into host motor circuits. This approach can harness the intrinsic neural plasticity to promote the integration of donor cells with host networks to drive meaningful and lasting connectivity and functional recovery.

Perhaps the most extensively studied ABT is locomotor training, either over-ground, treadmill, or device-assisted with robotics (e.g., Lokomat^®^). These strategies have been shown to promote plasticity and improve locomotor function across a wide range of SCI severities and spinal levels [267,268,269,270,271,272,273,274,275], both pre-clinically and clinically. Like other ABTs that have been explored (e.g., respiratory training), locomotor training leverages repetitive, task-specific movement to engage muscles and the spinal networks controlling them, strengthen residual motor output, and stimulate afferent feedback, thus driving related neural plasticity [276]. One of the key biological effects of this repetitive activity is the upregulation of trophic factors, such as BDNF, which are fundamental to neuroplasticity. Although locomotor training has historically been used to improve locomotion, it has also been shown to influence non-locomotor functions, including bladder control [277], cardiovascular function [278,279,280] and respiratory recovery [281], highlighting its broad neuromodulatory effects.

Preclinical studies combining ABTs with cell or tissue transplantation have demonstrated encouraging results. These combined approaches have been shown to: (i) prevent muscle atrophy downstream of denervated circuits [54,282], (ii) improve host motor neuron function [55,283], (iii) enhance growth of host SpINs into the transplant [284], and (iv) improve functional outcomes [285]. A variety of donor sources have been employed in these studies, including both FSC tissue and human PSC-derived neural stem/progenitor cells, with most efforts focused on hindlimb functional recovery following thoracic or lumbar SCI [50,55,282,286,287]. These findings underscore the potential for activity-based interventions to enhance transplant-host integration and amplify the therapeutic efficacy of transplanted cells.

Importantly, while ABTs rely on volitional or task-driven activation of neural circuits, other modalities of targeted neuromodulation—such as electrical, magnetic, or optical stimulation—offer an alternative means of driving activity-dependent plasticity, often at much higher resolution. Unlike ABTs, neural stimulation can more accurately target single muscle groups, nerves, or cell populations.

### 3.6. Neurostimulation

Neurostimulation refers to the application of electromagnetic energy that targets a specific neural structure. It is a subtype of neuromodulation, which broadly may refer to any technique that directly alters activity in a neural circuit, thereby affecting the bodily function(s) controlled by that neural circuit. Neurostimulation can have a therapeutic effect, a functional effect, or both. A therapeutic or rehabilitative effect is used to improve bodily function by applying neurostimulation over time and inducing physiological changes that persist even after the stimulation is discontinued. In this way, similar to ABTs, some neurostimulation approaches take advantage of the plasticity of the nervous system to improve or restore function. A functional or prosthetic effect is used to replace lost or impaired bodily function permanently.

When applying neurostimulation techniques, it is important to define the neural structure target that will affect the desired bodily function, the stimulation pattern that will modulate neural activity appropriately to achieve the desired modulation of bodily function, and the neural interface where the electrode (or other delivery device) makes contact with the neural structure. For example, epidural stimulation may use electrodes placed on the dorsal surface of the spinal cord and apply low-amplitude stimulation of sensory pathways to affect spinal circuits that promote improved control of the lower limbs. This approach can be combined with ABTs to achieve significant improvements in limb control over ABTs alone [288,289].

Neuromodulation approaches have been shown to leverage neuroplasticity to improve function after SCI. These approaches can facilitate motor re-engagement, stabilize synaptic connectivity, and promote circuit refinement through activity-dependent mechanisms. Evidence from both human and animal studies supports the therapeutic effects of neuromodulation in enhancing recovery [85,290]. However, these strategies are fundamentally limited by the availability and integrity of spared host neural networks—making their impact in severe injury cases variable and sometimes insufficient. That is, the greater the loss of neural circuitry following disease or trauma, the weaker the potential effects of stimulation will be when trying to modulate those remaining neural circuits.

Combining neurostimulation with cell therapy presents a unique opportunity to restore function by leveraging the strengths of each: the reparative potential of transplanted cells and the training potential of neurostimulation. The implantation of donor cells could provide additional neural substrate for neuromodulation, and the administration of neurostimulation could improve the integration of donor cells in the host. The electric field generated by electrical stimulation has been shown to affect the proliferation, migration, and differentiation of neural stem cells [164,291,292]. Recent work combined cell-containing scaffolds with epidural stimulation-enabled motor training in spinalized rats and found that newly regenerated axons through the scaffold reorganized neural circuitry below the lesion, and the rats showed improved motor recovery [293]. This in vivo experiment demonstrates that neuroregenerative and neuromodulatory therapies can synergistically enhance motor function after complete SCI.

Several factors must be considered when designing a combination strategy with neurostimulation and cell therapy, such as stimulation site, stimulation method and parameters, timing of combined approaches, and clinical feasibility. Neurostimulation may be applied directly to the site where the donor cells were introduced, or to neurons that can form connections to those donor cells. The location of the neural structure and the electrode used will also determine the specificity with which stimulation is applied. Several methods of stimulation can be applied, such as electrical, optical, or ultrasound, each with different possible patterns and parameters of stimulation, that can directly activate, promote activation, or inhibit neural activity. Stimulation parameters typically include intensity, frequency, and duration. These parameters should be chosen not only to achieve the desired effect, but also to avoid unwanted off-target effects, overstimulation and fatigue, and excitotoxicity. Neurostimulation should also be applied within an appropriate window of time. Some strategies may benefit from synchronizing stimulation with other events, such as movements during ABTs, or administering stimulation to coincide with peak plasticity post-cell transplantation. Finally, when considering which types of neurostimulation to use in combination with cell therapy, it is advised to choose neurostimulation approaches that have already been shown to be clinically effective. Several types of neurostimulation for different indications are clinically available, such as vagus nerve stimulation for epilepsy [294] or depression [295,296], phrenic stimulation for diaphragm pacing [297,298], epidural stimulation for pain or motor function [85,299], and sacral neuromodulation for bladder function [300]. These neurostimulation approaches are prime candidates to be combined with other approaches, including both ABTs and cell transplantation, in order to achieve a functional outcome that is greater than the sum of the parts.

### 3.7. Integrating Artificial Intelligence

Artificial intelligence (AI), including machine learning (ML) approaches, is starting to offer transformative potential in improving cell transplantation strategies for neural repair. Advances in AI, data science, computational modeling and deep learning not only enhance our technical [301] and analytical capabilities, they also enable novel insights into naïve neural cell and network dynamics [302,303,304], complement diagnostic assessments of acute and sub-acute spinal cord injury in patients [305], and may begin to transform how experiments can be designed, interpreted, and applied to biologically relevant therapies, all at remarkable speeds. ML has given us insights into the types of cells and networks needed to accomplish function, how we can more effectively direct the differentiation and AI-guided selection of donor cells toward therapeutically relevant phenotypes [306,307,308], and how we can entrain neural networks for optimal functions that contribute to relevant behaviors. There is an urgent need to compile data sets to support ML databases. The more information the field can provide ML platforms, the more accurate and informative the output will be from these systems. By integrating AI and ML into research, development, and clinical application of cell transplantation, we can accelerate progress toward effective neural repair therapies while reducing costs, improving outcomes, and minimizing risks [309].

As we seek to combine non-cellular treatments with cell transplantation, these same tools can be used to refine how we apply the combinations. AI-based tools are not only helping to refine the methods for engineering cellular therapies, but also the engineering of suitable biomaterials that can be combined with cells for transplantation [310,311]. ML can help to select the parameters for neural stimulation, drug delivery or ABTs, along with where and when to apply these non-cellular treatments relative to cell delivery [312]. As AI and ML become more integrated with health care, it will also become easier to identify patients that will benefit most from treatments, while also facilitating designer therapies with personalized treatment combinations. AI will also be able to analyze data from clinical trials to unbiasedly detect subtle patterns and correlations that might be missed by traditional methods, providing more effective evaluations of treatment outcome [312,313].

## 4. Closing Remarks

As the field evolves, emphasis will shift from single-modality interventions toward personalized combination therapies. Preclinical work already suggests that neural transplants show improved survival when paired with pharmacological, biomaterial, or other cellular support, and they integrate more effectively when combined with extrinsic guidance cues and host network activation. Future studies should systematically optimize these “cell + support + cue + activity” paradigms, for example, by deploying programmable or self-assembling scaffolds that release trophic and chemotropic factors over time, or integrating closed-loop neuromodulation systems that deliver stimulation in response to the maturation state of donor neurons. ML algorithms may prove invaluable for designing and scheduling such complex strategies, predicting which combinations (e.g., growth factors plus locomotor training plus epidural stimulation) best match a given injury level and severity, and determining the optimal timing and site for delivery for each component.

As our understanding of injury heterogeneity improves, and the contribution of neural cell phenotypes to repair and recovery progresses, customized transplants guided by patient-specific omics and imaging will become critical to ensure appropriate donor cell identity. Vascularized spinal organoids containing neurons, astrocytes, oligodendrocytes, and microglia may enable more complete circuit reconstruction, though advances in scaling, innervation, and immune compatibility are needed. These emerging neural-engineering and organoid-based transplants could synergize with combination approaches that can be tailored for personalized medicine. Ultimately, integrating diverse approaches—cells, biomaterials, molecular cues, rehabilitation and stimulation, and data-driven personalization—will be essential to harness endogenous plasticity and direct donor cells to form growth-supportive functional relays across injured spinal circuits offering lasting functional improvement.

A next-generation clinical trial for SCI could combine several emerging advances: donor cells that are enriched for region-specific reparative cell phenotypes (e.g., Chx10 or VSX1/2 expressing V2a spinal interneurons and their progenitors), modified to be hypo-immunogenic, delivered within a supportive and/or conductive biomaterial, followed by stimulation and/or intensive rehabilitation to shape emerging circuits. This type of combination therapy trial would directly target the major barriers to successful translation—immune compatibility, reparative donor cell phenotypes, donor survival, circuit engagement, and long-term stability—and offer a pathway toward testing truly integrated, multi-modal repair strategies.

## Data Availability

No new data were created or analyzed in this study.

## Abbreviations

The following abbreviations are used in this manuscript:

ABT	Activity-based therapy
AI	Artificial intelligence
BDNF	Brain-derived neurotrophic factor
cAMP	Cyclic adenosine monophosphate
CRISPR	Clustered regularly interspaced short palindromic repeats
CSPG	chondroitin sulphate proteoglycan
DREADDs	Designer receptors exclusively activatable by designer drugs
ECM	Extracellular matrix
FSC	Fetal spinal cord
hESC	Human embryonic stem cell
HLA	Human leukocyte antigen
iPSC	Induced pluripotent stem cell
MSC	Mesenchymal stem cell
ML	Machine learning
NPC	Neural progenitor cell
NT3	Neurotrophin-3
NSC	Neural stem cell
OEC	Olfactory ensheathing cell
OPC	Oligodendrocyte progenitor cell
PEDOT	poly (3,4-ethylenedioxythiophene)
PEG	Polyethylene glycol
PLGA	Poly lactic-co-glycolic acid
PSS	Polystyrene sulfonate
PSC	Pluripotent stem cell
SCI	Spinal cord injury
SpIN	Spinal interneuron
